# In vitro models of valproic acid to assess neurodevelopmental toxicity: A scoping review

**DOI:** 10.1111/epi.18392

**Published:** 2025-03-28

**Authors:** Daniel Sandvik, Elena Vianca, Alison Anderson, Muhammad Shahid Javaid, Terence J. O'Brien, Ana Antonic‐Baker

**Affiliations:** ^1^ Department of Neuroscience, School of Translational Medicine Monash University Melbourne Victoria Australia; ^2^ Faculty of Medicine University of Indonesia Jakarta Indonesia; ^3^ Department of Neurology Royal Melbourne Hospital Melbourne Victoria Australia; ^4^ Department of Neurology Alfred Health Melbourne Victoria Australia

**Keywords:** neurotoxicity, stem cell, teratogenicity, valproate, VPA

## Abstract

Valproic acid (VPA) is a first‐line antiseizure medication (ASM) that is highly efficacious for treating generalized and focal epilepsy disorders. Unfortunately, due to its strong association with teratogenic effects culminating in fetal valproate spectrum disorder (FVSD), which may include neurocognitive and neurobehavioral deficits, the drug has become highly regulated/restricted for women of childbearing potential. This includes those who have been shown to respond well to the drug and respond poorly to alternative ASMs. Concurrently, VPA's neurotoxic, teratogenic mechanisms have been studied in vitro, and continued research may aid in providing depth to our understanding so that superior evidence‐based care plans and novel drug designs can be made for patients with epilepsy disorders. This scoping review systematically assesses what in vitro studies have discovered regarding VPA's effects on neural cells and the proposed cellular neurotoxic mechanisms. Neurotoxicity studies have captured the cytotoxic, dysmorphological, genetic, and epigenetic effects in murine and human primary, immortalized, and stem cells in vitro. This includes extensive identification of many genes and gene pathways associated with neurodevelopmental disorders, a hallmark of FVSD. Although published studies have illuminated much about VPA's neurotoxic, teratogenic effects, a lack of standardization in testing methodologies renders making direct comparisons between the results of different studies challenging. Nevertheless, the recent use of human stem cell‐based models provides a richer understanding of what cellular, molecular, genetic, and epigenetic effects are caused by VPA exposure. Future in vitro studies may improve their clinical translatability by administering clinically relevant concentrations of VPA to human stem cell‐derived neural cells and fostering a better understanding of VPA's neural cell type‐specific and epigenetic effects. In vitro VPA neurotoxicity studies on neurodevelopment show a clear potential to provide data that may help construct superior personalized evidence‐based treatment plans and novel drug designs for women of childbearing potential with epilepsy disorders.

**Key points**

- VPA is one of the most highly efficacious first‐line ASMs that can treat multiple forms of epilepsy.
- Prenatal VPA exposure is associated with teratogenicity, rendering it contraindicated for women of childbearing potential.
- Some women of childbearing potential respond best to VPA over other ASMs.
- Studies using cell models in vitro have demonstrated that VPA causes a plethora of cellular and molecular effects.
- Future in vitro studies may improve their testing methodology to better understand the neurotoxic, teratogenic mechanisms of VPA.

## 1 INTRODUCTION

Valproic acid (VPA) is a commonly prescribed and efficacious first‐line antiseizure medication (ASM),^1^ and it is used to treat children and adults with generalized and focal epilepsy, among several other nonepileptic conditions.^2^ The drug's modes of action are yet to be fully understood. Still, some proposed mechanisms that attempt to explain VPA's antiseizure effects include increasing the amount of the inhibitory neurotransmitter γ‐aminobutyric acid in the brain^3^, ^4^ and modulating sodium and calcium ion channel influx and efflux.^5^, ^6^, ^7^ VPA is also a histone deacetylase (HDAC) inhibitor that can prevent the removal of acetyl groups on histones, which increases the accessibility of DNA and the rate of RNA transcription.^8^

Despite being highly efficacious for treating epilepsy and having a favorable tolerability profile,^9^ VPA is generally considered to be contraindicated for women of childbearing potential unless there are no other effective alternatives due to its strong association with causing birth defects.^10^, ^11^ Although structural defects have long been observed in VPA teratogenicity studies, its neurotoxic effects on neurodevelopment have become a concern in recent years. Prenatal VPA exposure increases the risk of neurodevelopmental disorders in children, such as autism spectrum disorder (ASD),^12^, ^13^ attention‐deficit/hyperactivity disorder,^14^ and neurocognitive deficits.^15^ Despite the risks of teratogenicity, some women's seizures respond best to VPA compared to alternative ASMs, in particular those with generalized epilepsies.^11^, ^16^ Therefore, it is likely that some women are being denied the most effective epilepsy treatment due to VPA's strict regulations and concerns about teratogenicity and neurodevelopmental effects on the unborn child.

To construct well‐informed care plans for patients with epilepsy, the mechanisms by which VPA causes developmental issues should be investigated. Some implicated neurotoxic mechanisms include mitochondrial dysfunction leading to oxidative stress,^17^ disruption of folic acid transport and metabolism for the fetus leading to increased risk of neural tube defects and ASD,^18^ and the inhibition of HDACs that interact with histone and nonhistone proteins affecting neuronal maturation and neural tube closure.^19^ However, despite decades of research, the precise mechanisms have not been elucidated. Many of these neurotoxic mechanisms have been elucidated from in vivo and in vitro animal and human studies. However, each model type presents its own set of advantages and disadvantages.

This scoping review assesses the cellular and molecular neurotoxic mechanisms of VPA, followed by a discussion of each model's strengths and limitations. Finally, we will present possible future directions of using neural cells in vitro to research VPA's neurotoxic mechanisms and relate how improving our understanding of said mechanisms may translate into providing better treatments for patients with epilepsy disorders.

## 2 MATERIALS AND METHODS

This review used the PRISMA‐ScR (Preferred Reporting Items for Scoping Review) guidelines.^20^ We utilized rigorous systematic review methodologies to minimize selection bias.

### 2.1 Search strategies

We searched the Web of Science, MEDLINE, and Embase electronic databases. Our search terms were as follows.

- Search terms for VPA: “Valproic acid” or “Sodium valproate” or “2‐propylvaleric acid” or “Valproate semisodium” or “Depakote” or “Epilim” or “Convulex”.
- Search terms for teratogens: “Teratogen” or “Neurotoxin”.
- Search terms for in vitro models: “In vitro” or “In vitro techniques” or “Cell culture” or “Culture”.

Appropriate wildcard search symbols and Boolean operators were used.

### 2.2 Eligibility criteria

We included all original studies that used in vitro models derived from any mammalian organism to study VPA's effect on neural cells. The literature search was performed in February 2024. No year limit was set, as we wanted to assess all available articles related to the progress of VPA research. We excluded nonprimary articles such as letters, reviews, books, abstracts, and conferences, as well as in vivo studies, clinical trials, studies investigating the neuroprotective effects of VPA, and studies not using neural cells.

### 2.3 Publication selection

All publications were screened under our aforementioned inclusion and exclusion criteria. The publications were screened by title and abstract, followed by full‐text by at least two independent reviewers (D.S., E.V., and M.S.J.). Discrepancies between reviewers were resolved by another reviewer (A.A.‐B.).

Information was extracted using a standard data extraction template that categorized the studies by species, cell type, differentiation protocol (where relevant), concentration, time of and length of time of VPA administration, duration of experiments, and primary outcomes (Table S1). All the retrieved publications were managed with Covidence software.

### 2.4 Information extraction

Data were extracted from the included scoping review publications by two independent reviewers (D.S. and E.V.) using a predetermined data extraction Excel form. Any disagreements were resolved with discussion and an additional reviewer (A.A.‐B.). Extracted data included species, cell type, differentiation protocol (where relevant), concentration, time of and length of time of VPA administration, duration of experiments, and primary outcomes (Table S1).

## 3 RESULTS

### 3.1 Study inclusion

**FIGURE 1:**
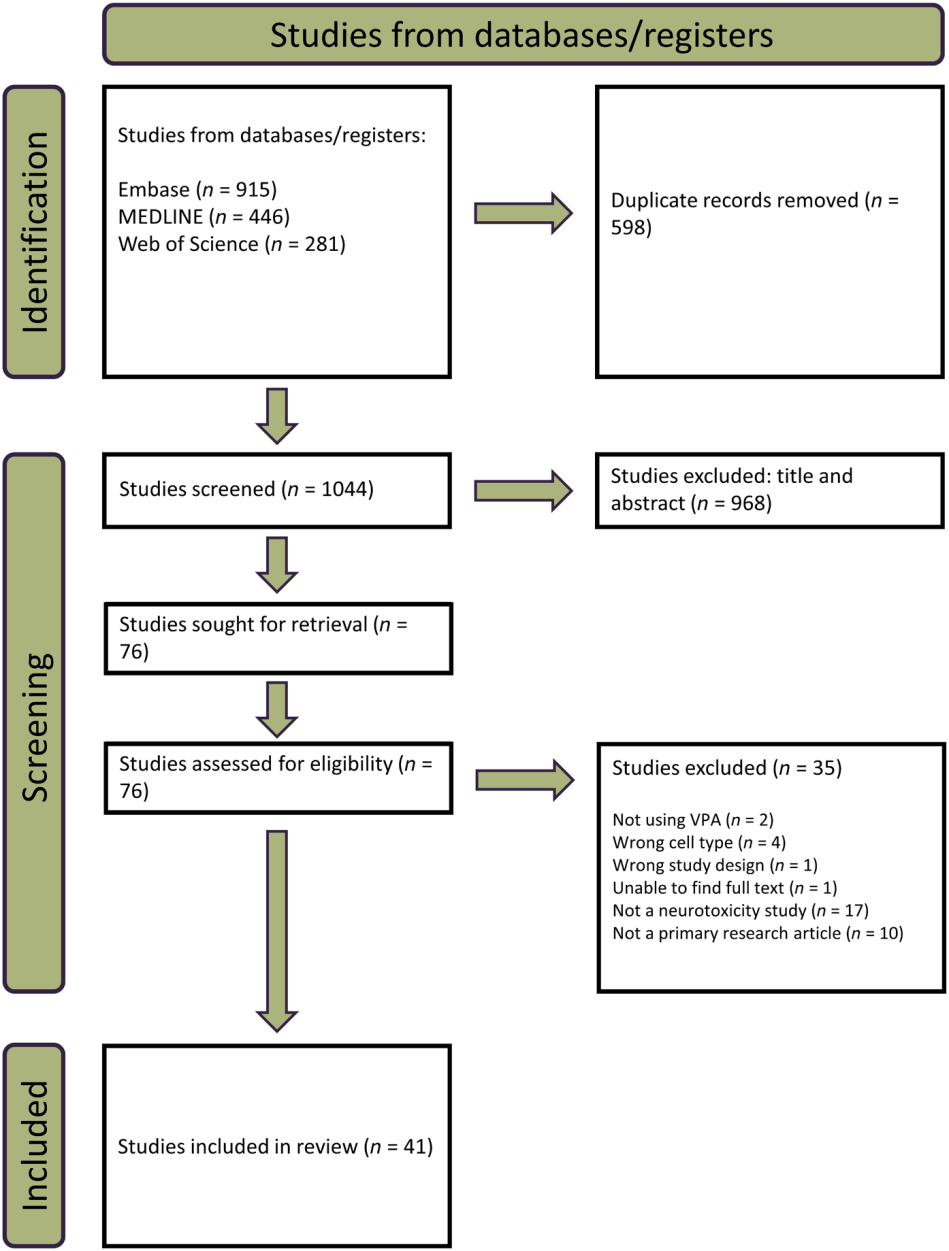
PRISMA (Preferred Reporting Items for Systematic Reviews and Meta‐Analyses) flowchart of database searches, two‐phase screening, and data extraction. Of the 1642 studies, 1031 were screened for title and abstracts. Of these, 76 studies underwent full‐text screening, identifying the 41 studies spanning 1988–2023 included in this review. VPA, valproic acid.

**FIGURE 2:**
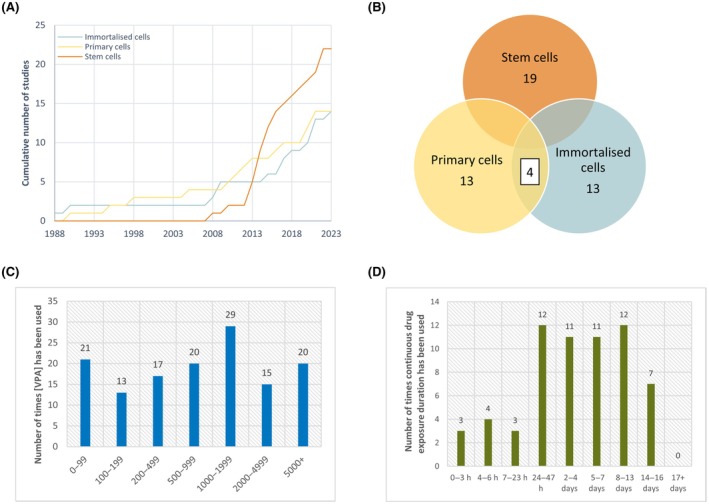
Parameters of the extracted in vitro valproic acid (VPA) neurotoxicity studies. (A) The cumulative frequency of primary cell, immortalized cell, and pluripotent stem cell studies investigating the neurotoxic effects of VPA from 1988 to 2023. (B) Venn diagram shows the number of studies extracted by cell type, including studies that used multiple cell types. (C) Number of times specific VPA concentrations were used throughout the literature. (D) Number of times specific durations of VPA exposure were used throughout the literature. Total studies = 41 (19 stem cells, 13 primary tissues, 13 immortalized cells).

A total of 1642 studies were identified, with 41 meeting our inclusion criteria after screening (Figure 1, Table S1). The included studies were categorized into three main cell types: primary cells, immortalized cells, and stem cells of human or murine origin. More research was published in the 2010s compared to the 1980s–2000s, with stem cell research demonstrating more rapid growth than immortalized or primary cell studies (Figure 2A).

Of the included studies, 19 used stem cells, which can differentiate into various cell types. At the same time, 13 studies used primary or immortalized cells, with four using both primary and immortalized cells (Figure 2B). The most commonly used concentration of VPA in vitro is 1000–2000 μmol·L^−1^ (Figure 2C), and the most frequently used durations of continuous VPA exposure are approximately 24 h or between 1 and 2 weeks. None of the studies used VPA beyond 16 days (Figure 2D). Commonly measured endpoints included cell morphology and viability, mitogenesis, mitochondrial function and oxidative stress, and gene and protein expression.

In 1988, for the first time, Martin et al.^21^ established VPA's effects on neuronal mitosis and cell differentiation using immortalized murine cells. The first identified human stem cell study in 2008 determined VPA's half‐maximal effective concentration for cell viability.^22^

### 3.2 Cellular effects

#### 3.2.1 Cytotoxicity: VPA exposure severely affects cell viability at ≥1000 μmol·L^−1^

In 1998, Fennrich et al., administered 5000 μmol·L^−1^ VPA to primary rat neurons for 6–24 h and observed an increase in apoptotic markers.^23^ The expressions of cleaved caspase‐3 and poly(adenosine diphosphate‐ribose) polymerase (PARP) and the reduction of nuclear factor kappa B (NF‐κB) DNA binding combined with the degradation of p65 have all been recorded.^24^, ^25^ Three studies demonstrated a dose‐dependent decline (.0001–10 000 μmol·L^−1^ for 1–12 days) in neural cell numbers for primary rodent cerebellar granule cells, neural progenitor cells, and SH‐SY5Y cells.^4^, ^26^, ^27^ Particularly for SH‐SY5Y cells, VPA (500–1500 μmol·L^−1^ for 2 days) appeared to decrease cell adhesion and increase cell detachment.^21^

Hansen et al. have explored several mechanisms underlying cell death. The simultaneous production of reactive oxygen species and reduced activity of the antioxidant enzyme superoxide dismutase were observed in both primary CD‐1 mice and immortalized P19 cells. Although VPA is not an oxidant, the authors noted it can influence oxidative processes within neural environments.^28^ Oxidative damage occurred in primary Wistar rat neural cells, where increased protein carbonyl content and decreased antioxidant enzyme activity were measured.^29^

A study using SH‐SY5Y and primary mouse cerebellar granule (LAN‐1) cells demonstrated increased expression of P75 neurotrophin receptor (p75NTR) and sortilin, peaking at 9 h of 1000 μmol·L^−1^ VPA exposure. Pro‐nerve growth factor (ProNGF)‐mediated c‐Jun N‐terminal kinase (JNK) activation and apoptosis were also noted. Combined, there was evidence of VPA mediating cell survival.^30^ Oxidative stress and apoptosis were also present in another SH‐SY5Y cell study, where the addition of melatonin reduced these adverse effects on cell survival.^31^ Astrocytes isolated from 12‐week‐old aborted human fetuses exposed to VPA (250–1000 μmol·L^−1^ for 7 days) released tumor necrosis factor α, which likely caused the death of Tuj1‐positive or βIII‐tubulin‐positive neurons.^32^

Evidence suggests that VPA's impact on cell viability varies across cell types and maturation stages. Neuronal cell viability, dendrite arborization, and synapse maturation of immature neurons derived from H9 human embryonic stem cells (hESCs) decrease from exposure to ≥800 μmol·L^−1^ of VPA for 3–14 days. In contrast, mature neurons do not exhibit these effects,^33^, ^34^ potentially due to VPA activating prosurvival signaling patwhays.^25^ At concentrations of VPA >1000 μmol·L^−1^, human induced pluripotent stem cell (hiPSC) viability decreased. In comparison, exposure to lower concentrations (between 70 and 210 μmol·L^−1^) of VPA for 3 days increased the number of neurites per neuron,^35^ contrary to the decreased number seen in primary rat cerebellar granule cells.^27^

#### 3.2.2 Dysmorphology: VPA disrupts the morphologies of neurons and glia in all three main cell types across murine and human models

Early literature observed VPA‐induced (10–5000 μmol·L^−1^ for 2–14 days) morphological changes in immortalized N2a and C6 mouse cells and primary rat cells, such as aberrant branching processes, inappropriate migration, degraded cell integrity, and cytotoxic effects.^21^, ^23^, ^36^ VPA at 1000 μmol·L^−1^ also downregulates the expressions of docking protein 6, where respective gene knockout experiments in mice caused decreased neurite outgrowth.^37^

In contrast, astrocytes exposed to 50–5000 μmol·L^−1^ of VPA for 14 days misroute, reduce in cell length, randomize, and distort their processes.^23^ Neurospheres grown from aborted human fetuses exposed to VPA (250–2500 μmol·L^−1^ for 6–10 days) reduced both in size and neuronal process length. Outer radial glia associated with human dorsal forebrain neuronal stem cells exposed to VPA (100–500 μmol·L^−1^ for 12 days) induced disorganized cortical lamination and the loss of upper layer neurons.^38^ Radial astrocytes extracted from primary rat forebrain and hippocampus tissues exposed to 50–5000 μmol·L^−1^ of VPA for 14 days exhibited reduced astrocytic process length and misrouting. Neurons also accumulated abnormally, and the spatial orientation of glial cell processes was randomized and distorted.^23^

### 3.3 Molecular effects

VPA induces broad alterations in gene and protein expression, as evidenced by studies employing transcriptomics.^34^, ^39^, ^40^, ^41^ These studies have identified that VPA deregulates the expressions of hundreds of genes simultaneously across multiple cell types and between murine models and human samples. However, directly comparing the results of each study is difficult due to variations in VPA concentrations, exposure durations, and cell types used. Nonetheless, evidence of molecular effects will be presented here.

#### 3.3.1 Epigenetic mechanisms: VPA regulating HDAC activity underpins its widespread gene‐dysregulating effects

Several in vitro studies using immortalized, primary, or stem cell models from human or murine samples demonstrate that VPA inhibits HDAC activity and deregulates the expressions of many factors that interact with HDAC genes.^30^, ^41^, ^42^, ^43^ Furthermore, known HDAC inhibitors such as trichostatin A and entinostat mimicked effects seen in human SH‐SY5Y immortalized cells or mouse LAN‐1 primary cells exposed to 1000 μmol·L^−1^ of VPA for 24 h.^30^ Konala et al.,^42^ confirmed that class I HDACs are specifically inhibited by VPA administered to human stem cells at concentrations between 1000 and 80 000 μmol·L^−1^ for 15 days. Therefore, it has been proposed that one of VPA's primary neurotoxic mechanisms of action is to deregulate many genes simultaneously.^19^

#### 3.3.2 Impact on cell type markers: VPA causes dose‐dependent effects on the expression of stem cell genes

In H9 hESCs, treatment with 33–1000 μmol·L^−1^ VPA for 7 days significantly decreased the expressions of *OCT4*, *NANOG*, *TUBB3*, *NEUROG1*, *RELN*, *MAP2*, and *MAPT* in a dose‐dependent manner.^44^ Conversely, hiPSCs exposed to 500 μmol·L^−1^ of VPA for 9–19 days showed increased *OCT4*, *NANOG*, and *SOX1* expression with decreased *PAX6* expression.^45^ Another H9 cell study confirmed the VPA‐induced regulation of *SOX1* (25–1000 μmol·L^−1^).^46^ Higher VPA concentrations (1000–80 000 μmol·L^−1^) administered for 15 days showed increased *OCT4* and *PAX6* expression while decreasing *SOX2* and *MAP2* expression.^42^

Human neural progenitor cells dosed with 250 μmol·L^−1^ of VPA for 5–10 days showed increased expressions of neuronal cell body genes *TUBB3* and *MAP2*, synaptic markers such as *SYNPR* and *PSD95*, and vesicle markers such as *VGLUT2*.^47^ MicroRNA and gene expression analysis of mouse stem cells exposed to 300 μmol·L^−1^ of VPA for 16 days observed downregulation of neural markers and upregulation of myogenic markers.^48^ Additionally, the expression of astrocyte marker *GFAP* and total cellular protein in primary human fetal neural stem cells decreased dose‐ and time‐dependently following 250–2500 μmol·L^−1^ of VPA administration.^49^

#### 3.3.3 Impact on neurodevelopmental markers: VPA exposure alters cell metabolism and expression of genes involved in neurodevelopment

H9 cells exposed to 100–1000 μmol·L^−1^ of VPA for 24 h dysregulated *TUBB3*, *HOXA1*, *EPHA2*, *MAP2*, *NODAL*, *FGF8*, *MYC*, *CTGF*, and *COL1A2*. These genes encompass neuron differentiation, nervous system regulation, neural plate development, neurofilament cytoskeleton organization, cell morphogenesis, ectodermal development, or axis and pattern formation.^39^ H9 cells exposed to VPA at concentrations > 90.8 μmol·L^−1^ demonstrated negatively affected ornithine metabolism, a marker of cell growth and protein synthesis.^50^ In contrast, immortalized rat NS‐1 cells (a subclone of PC12 cells) exposed to .001–100 μmol·L^−1^ did not have their growth affected by VPA.^51^ SH‐SY5Y cells exposed to up to 1000 μmol·L^−1^ of VPA for up to 72 h exhibited an increased expression of *SCN1A* and *SLC7A5*.^52^ Immortalized mouse C17.2 cells exposed to 100 μmol·L^−1^ VPA for 5 days showed upregulated expression of *LYNX1* and downregulated expression of *OLIG2*, *SEMA5B*, *GABRB2*, and *SLC6A7*.^27^ In P19 immortalized cells, there was downregulation of *FOLR1* and *MARCKSL1* (VPA 500–25 000 μmol·L^−1^ for 1.5–24 h).^19^ A separate H1 and H9 cell study confirmed that a 3–14‐day exposure to 1000 μmol·L^−1^ of VPA decreased MARCKSL1 expression and induced cellular changes akin to neural tube defects that were rescued by overexpression.^33^

#### 3.3.4 Impact on cellular differentiation: VPA effect on expression of genes and proteins involved in cell differentiation processes

SH‐SY5Y cells exposed to 75 μmol·L^−1^ of VPA for 6 days had the expressions of the axonal outgrowth genes *NTNG2*, *NRXN1*, and *SEMA5A* downregulated.^26^ These results conflict with an observed upregulation of *NRXN1* and downregulation of *SEMA5A* in H9 stem cells with 500–2000 μmol·L^−1^ of VPA exposure for 6–14 days.^41^ VPA in concentrations between 139 and 555 μmol·L^−1^ administered to rat fetal telencephalon tissues showed an increase in choline acetyltransferase, glutamic acid decarboxylase, and 2′,3′‐cyclic nucleotide 3′ phosphohydrolase activity',^53^ potentially indicating differentiation‐enhancing effects. PC12 cells given 25 000 or 50 000 μmol·L^−1^ of VPA for 4 h exhibited increased *GATA3* expression, which has a role in brain development and cell differentiation.^54^

#### 3.3.5 Signaling pathways: VPA exposure disrupts signal pathways and epigenetic regulation

For H9 cells differentiated into neurons, VPA administration (100–1000 μmol·L^−1^ for 7 days) affected Wnt and transforming growth factor β (TGFβ) signaling, which regulates stem cell self‐renewal, neuroectoderm formation, cell proliferation, differentiation, and migration.^40^ The Erk pathway was also activated, which is responsible for cell growth, proliferation, survival, neuronal signal transduction, and neuron maturation.^40^ Other studies also showed that VPA regulates the expression of genes that interact with the Wnt/β‐catenin signaling pathway and the Shh (sonic hedgehog) signaling pathway, negatively affecting neurogenesis.^19^, ^38^, ^48^ PC12 cells exposed to 50–5000 μmol·L^−1^ of VPA for 3 or 5 days demonstrated significant cell death via necroptosis induced by calpain signaling activation.^55^

Gene ontology (GO) term enrichment analyses have implicated various biological functions in VPA toxicity. These include ion transport, synaptic function, axon development, calcium ion binding, chromatin and histone modification, neural processes, developmental processes, DNA and RNA processing, neural tube formation, embryonic morphogenesis, stem cell development, mitosis, apoptosis, nervous system development, and transcriptional regulation. These studies used human and murine stem cells dosed with VPA at 3–2000‐μmol·L^−1^ concentrations for 1–14 days.^34^, ^39^, ^40^, ^41^, ^47^, ^56^ Two studies enhanced their GO term analyses by verifying VPA's interactions with transcription factor binding sites (TFBSs).^34^, ^41^ Waldmann et al.^34^ noted that the number of differentially regulated genes, identified GO groups, and TFBSs was increased dose‐dependently with VPA concentrations > 25 μmol·L^−1^ but mostly up to 350–450 μmol·L^−1^. In a later study, the team proposed that VPA's alteration of epigenetic states via HDAC inhibition may lead to widespread transcriptomic changes that may underpin cytotoxic and noncytotoxic effects.^46^

#### 3.3.6 Disease‐related genes and pathways: VPA exposure affects gene and protein expression involved with neurodevelopmental disorders

PC12 cells administered with 25 000 or 50 000 μmol·L^−1^ VPA for 4 h caused increased expression of the transcription factor *GATA3*. This transcription factor is expressed in serotonergic and dopaminergic neurons and is involved with neurotransmitter synthesis, with disturbances to its expression associated with noise sensitivity in ASD. Therefore, VPA‐enhancing *GATA3* activity is posited to cause excessive differentiation of serotonergic and dopaminergic neurons alongside neurons involved in sound perception, as seen in ASD.^54^ An H9 cell study observed decreased asymmetric dimethylarginine (ADMA) after 1154 μmol·L^−1^ VPA exposure for 4 days. ADMA is a metabolite required for neural tube closure, and in utero VPA exposure is often attributed to abnormalities in neural tube closure.^57^ VPA (10–5000 μmol·L^−1^) added to primary 7‐day‐old rat pup cerebellar granule cells for 24 h decreased SNAP‐25 protein expression by up to 27% in a dose‐dependent manner. This protein is involved with presynaptic intracellular vesicular trafficking and exocytosis, and decreases in this protein are associated with hyperactivity and cognitive issues seen in neurodevelopmental disorders.^58^ In a rat model, 600–5000 μmol·L^−1^ of VPA exposure for 24 h to primary neural progenitor cells increased the expression of T‐type calcium channels. This was presumed to be due to VPA‐mediated inhibition of histone deacetylase activity based on increased acetylation of histone H3 within the promoter region of the *CACNA1G* gene, which encodes the CaV3.1 channel. The authors suggest that the upregulation of T‐type calcium channels contributes to the development of neurodevelopmental disorders.^6^ A stem cell study using H9 and HUES1 hESCs showed that VPA (3–1250 μmol·L^−1^ for 9 days) deregulated the neural tube and neural plate genes *EPHA7* and *TEX15*, associated with neural tube defects.^56^

## 4 DISCUSSION

This scoping review demonstrates that studies using in vitro cell‐based models have provided considerable insight into the myriad cellular and molecular neurotoxic/neurodevelopmental effects caused by VPA exposure in utero. These findings generally include VPA contributing to reduced cell survival, reduced cell growth, and the promotion of apoptotic markers while disrupting the expression of many genes and the function of epigenetic mechanisms. Although in vitro models present some clear advantages for investigating particular research questions, other paradigms may be suitable for answering different questions. Here, we will present the pros and cons of using in vitro models to study VPA's neurotoxic mechanisms and suggest what research questions could be investigated in the future to address the treatment of patients with epilepsy better.

### 4.1 Comparison of the advantages and disadvantages of various in vitro models

Using in vitro models to study VPA neurotoxic/neurodevelopmental effects confers several advantages, including being less expensive and time‐consuming than in vivo animal models^59^ and the ability to use human‐derived cells to model human outcomes in an ethical manner.^60^ Organoid in vitro models, such as neurospheres, can mimic some three‐dimensional neural cell organization in vivo.^61^ Naturally, a goal of using stem cells in vitro is to differentiate them into a diverse range of neural cells in a way that is most similar to neurodevelopment in vivo. Various neural induction and differentiation models can achieve this. For example, the dual SMAD inhibition protocol used in some stem cell studies presented in this review^33^, ^34^, ^41^, ^45^, ^46^ can induce stem cells into a neural lineage. This is achieved by adding the small molecules SB‐421542 and LDN‐193189 to block the activation of the bone morphogenetic protein and TGFβ signal cascades that utilize SMAD proteins to specify the endoderm and mesoderm cell fates.^62^, ^63^ Then, depending on the cell type(s) of interest, neural cell differentiation is initiated by the addition of a certain combination of growth factors.^64^, ^65^ The dual SMAD inhibition protocol facilitates a diverse range of neural cells to be grown in vitro over a more gradual time akin to in utero development.^66^ In comparison, the use of nonhuman, non‐stem‐cell models raises questions regarding how accurately these models capture the effects of VPA on the expression of genes related to cell growth and axonal outgrowth. It is thus clear that, over time, human stem cells have become the in vitro model of choice for studying VPA's neurotoxic effects.

However, despite some studies using highly accurate neural cell induction protocols such as dual SMAD inhibition, no studies identified in this scoping review have attempted to culture stem cells beyond 56 days^33^ or 125 days^45^ or continuously exposed these cells to VPA for any longer than 16 days.^48^ Due to the need of longer culture times to grow glia, this means that very few studies have investigated the effects of VPA on the codevelopment of glia with neurons and what effects astrocytes may have on neuronal properties, for example.^45^ Of the few studies that investigated astrocytes and VPA exposure, it has been shown that VPA affects astrocyte structure and functioning,^4^, ^23^, ^32^, ^49^ while causing these glia to release factors that encourage the death of neurons.^32^ Nonetheless, most studies focus on the neurotoxic effects VPA has on neurons. Hence, we propose that the full potential of using human stem cell models is yet to be realized and could be achieved with more extended cultures that generate more diverse neural cell types. Furthermore, the correlation between in vitro models and human pregnancy was explored by Gordon et al.^67^ They determined that “three‐dimensional human cortical organoids reach postnatal stages between 250 and 300 days, paralleling in vivo development.” Thus, the effects of VPA on the development of first‐trimester neural cells can be accurately compared to second‐ or third‐trimester neural cells using in vitro models.

### 4.2 What is a “clinically relevant” VPA concentration that can be used in vitro?

Many studies have not exposed cells to VPA within the established average serum concentration range in patients (approximately 346–867 μmol·L^−1^),^10^ and it could be argued that doing so would more accurately model what occurs to fetal neural cells if a pregnant patient was using VPA. However, it is possible that a “serum concentration” of VPA is not what developing neural cells are exposed to in utero. Very few pharmacological studies have evaluated the proportion of VPA that passes through the mother to the fetal tissues. One 1979 case study established that after passing from the serum through the placenta, neonatal serum concentrations of VPA were higher than in the mother,^68^ indicating that using an average serum concentration of VPA in vitro is an underestimation. But a more recent 2021 study using pregnant rat dams and their progeny showed that although the proportion of VPA entering the neural tissues of the fetuses was higher than the mother's neural tissues, ultimately, only a fraction of the serum concentration was entering the fetal neural tissues.^69^ This potentially indicates that administering a concentration of VPA equivalent to maternal sera on developing neural cells in vitro may result in an overestimation of the drug's neurotoxic effects. It is here that the utility of in vivo animal studies becomes apparent, as more pharmacological research is required to establish a “pregnancy‐like” concentration of VPA that can be administered to cells in vitro over time, mainly due to the placenta and fetal blood–brain barrier being modulatory factors. It will be important to validate findings from in vitro transcriptomic studies using in vivo models, as cellular responses may differ in the presence of additional cell types or epigenetic mechanisms that are absent in the artificial environment. In addition, establishing the relative effects of peak exposure compared to the duration of exposure and constructing exposure‐dependent curves that graph VPA's adverse effects across models could help improve the translatability.

### 4.3 Future directions

The impact of genetic variation in risk of valproate teratogenicity remains to be fully understood, and in vitro stem cell research is well placed to address this challenge. Across individuals, a genetic predisposition is likely heterogenous and potentially multifactorial. Pharmacogenomic variants that modify the metabolism of the drug, or drug efflux,^70^ modify risk in terms of dose; in contrast, variants in genes that disrupt the regulation of cardiomyocyte or neuronal differentiation may be critical to risk of cardiac malformations or neurotoxicity, respectively. The perturbation of folate metabolism may occur due to genetic variation impacting on the structure of proteins within the folate metabolism pathways, or alternatively through disruption of mechanisms that regulate the expression of genes encoding these proteins, or both. In vitro studies provide opportunity to address this complexity with assays designed to tease out the cell type‐specific consequence of individual variants or variant combinations. CRISPR screening can, for instance, be applied to detect variants/genes relevant to specific adverse effects. Transcriptomics combined with chromatin assays such as transposase‐accessible chromatin with sequencing can provide insight into the epigenetics of teratogenicity, and isogenic lines that introduce variation into normal induced pluripotent stem cells or correct a patient‐derived line can determine variant and cell type‐specific functional consequences.^71^ By highlighting important regions of the DNA, where risk can be modified, variant panels for pretreatment tests can be developed and the toxicology screening of new drugs enhanced.

Future single‐cell and spatial transcriptomic studies that detect VPA's effects on chromatin dynamics will likely provide meaningful insights into how the drug affects HDACs, specific cell types, and widespread genetic and epigenetic dysregulation.^72^, ^73^, ^74^ The limited use of commercialized hiPSCs in the literature could be expanded to include patient‐derived hiPSCs, which may help assess genetic factors that influence the risk of teratogenic pregnancy outcomes.^75^ By leveraging these technologies, we may start to better understand some mechanisms that at least partially answer broad clinical questions such as “Why does VPA cause teratogenic effects in only a minority of pregnancies?”^76^ and “Why does VPA contribute to a spectrum of teratogenic and neurodevelopmental effects among affected children?”^77^

### 4.4 Strengths and limitations of our approach

The strengths of our study include having a broad scope to sample a large amount of literature across time from three databases while following strict systematic review guidelines regarding how we searched, screened, included, and excluded studies. Additionally, the size of our team minimizes the risk of bias in our screening process.

However, the robustness and confidence in our conclusions are mainly mitigated by the variability of the study aims, assays, cell types, and differentiation protocols. This lack of standardization between studies limits our ability to compare different studies' results directly. Moreover, our broad aim to assess how in vitro models have been used to study VPA's neurotoxic effects means we likely have not exhaustively covered how every model and each technique applied to each model can be used.

## 5 CONCLUSIONS

Through our systematic analysis of >35 years of relevant research, it has become clear that in vitro studies have provided, and have much more potential to continue providing, valuable information about VPA's neurotoxic and adverse neurodevelopmental mechanisms. Using in vitro models has demonstrated that VPA has significant cytotoxic, dysmorphological, genetic, and epigenetic effects, thus recapitulating clinical findings. Human stem cells offer considerable insight into how VPA regulates neural cell functions. Due to the broad advantages of using human stem cells, we assert that continuing to use these models while upgrading current methodologies may help answer many of the remaining research questions to form a better holistic understanding of how VPA's broad neurotoxic effects can be explained. Specifically, in vivo studies that establish the appropriate concentrations of VPA to account for fetal–maternal drug modulation mechanisms would aid in providing a “clinically relevant” dose in vitro. By furthering our understanding of the mechanisms of VPA's neurotoxicity, we are more likely to be well informed on how to create superior evidence‐based treatment plans for women of childbearing potential with epilepsy.

## AUTHOR CONTRIBUTIONS

**Daniel Sandvik:** Writing—original draft preparation (lead); writing—review & editing (equal); conceptualization (equal); investigation (equal); visualization (lead). **Elena Vianca:** Writing—review & editing (equal); investigation (equal). **Alison Anderson:** Writing—review & editing (equal); investigation (equal). **Muhammad Shahid Javaid:** Investigation (supporting). **Terence J. O'Brien:** Writing—review & editing (equal); funding acquisition. **Ana Antonic‐Baker:** Conceptualization (equal); investigation (equal); writing—review & editing (equal).

## CONFLICT OF INTEREST STATEMENT

The authors declare no conflict of interest. We confirm that we have read the Journal's position on issues involved in ethical publication and affirm that this report is consistent with those guidelines.

## Supporting information


Table S1.

## Data Availability

The data that supports the findings of this study are available in the supplementary material of this article.
