# Supplementary material for: In vitro models of valproic acid to assess neurodevelopmental toxicity: A scoping review
Source: Epilepsia. 2025 Mar 28;66(7):2170–81. doi: 10.1111/epi.18392 (PMC12291010; doi:10.1111/epi.18392)
Supplement: Supplementary file 1 — Table S1. [file EPI-66-2170-s001.docx]

**Table S1.** Data extracted from the included studies that tested neurotoxicity caused by VPA in cells *in vitro*^1–41^.

| **Author** | **Year** | **Species** | **Cell type** | **[VPA] in µM** | **Duration of VPA exposure** | **Outcome Measure** | **Main Outcomes** |
| --- | --- | --- | --- | --- | --- | --- | --- |
| **Attoff** | **2017** | Mouse | Immortalised (C17.2) | 0.001 to 10,000 | 5 or 10 days | Gene expression, cell viability | Cell Viability: Decreased to 50% between 1000 and 10,000 uM VPA  Neuronal Morphology: Lowered the number of neuronal cells; Reduced neurites per neuron  Gene Expression Changes (10 days after differentiation, 100 μM VPA):   Most upregulated gene: LYNX1  Most downregulated genes: OLIG2, SEMA5B, GABRR2, SLC6A7 |
| **Martin** | **1988** | Mouse and rat | Immortalised (N2a and C6) | 0, 500, 1000, 1500 | 48 hr | Mitotic rate, cell adhesivity, cell differentiation | VPA effects on glioma and neuroblastoma cells at ≥500μM:  Mitotic rate: Significantly decreased in both cell types  Cell-substratum adhesivity: Increased in glioma cells  Cell differentiation: Induced in neuroblastoma cells |
| **Regan** | **1990** | Mouse in vivo, mouse in vitro, rat in vitro | Immortalised (N2a and C6) and primary | 0, 100, 500, 1000, 1500, 2000, 3000 | 48 hr | Antiproliferative effects, cell cytotoxicity | Antimitotic IC50 concentrations for C6 and N2a respectively were 1300 and 1200 µM VPA; cytotoxicity was observed at 2 days at 3000 µM; exencephaly was only observed in mouse embryos (species selectivity). |
| **Radio** | **2008** | Rat | Immortalised (NS-1) | 0.001 to 100 | 96 hr | Neurite outgrowth, cell viability | Valproate did not increase PC12 cell growth with exposure to 0.001 to 100 uM of VPA. |
| **Jergil** | **2009** | Mouse | Immortalised (P19) | 0, 500, 1000, 2500, 5000, 10000, 25000 | 1.5, 6 or 24 hr | Cell viability, apoptosis, gene expression | 68 downregulated and 125 upregulated genes  VPA effects on the cell cycle and apoptosis: Antiproliferative effects at 1000-2500 μM, causing G1 arrest; Activation of cleaved caspase 3 indicating apoptosis; G1-S phase apoptosis genes identified in microarray  VPA effects on HDAC Inhibition: HDAC target genes affected: Lefty1 (downregulated), Bmp4 (upregulated)  VPA effects on Signaling Pathways: Sonic hedgehog (Shh); Wnt; Retinoic acid  Other VPA-affected Gene Categories: Transcription factors; Histone acetylation-related genes; Zinc-binding proteins |
| **Lamparter** | **2017** | Mouse | Immortalised (P19) | 5000 | 24 hr | Gene and protein expression, DNA binding | Protein and mRNA Expression: Total p65 and p50 protein and mRNA expression downregulated; NF-κB DNA binding reduced  NF-κB Activity: Overall increased NF-κB transcription activity; Decreased p65 phosphorylation at ser276  Mechanisms: PTMs, including ser276 phosphorylation, protect p65 from ubiquitination and degradation; Caspase activation can degrade p65; VPA may increase NF-κB activity by inhibiting HDACs, reducing p65 acetylation |
| **Hansen** | **2021** | Mice (CD-1) | Immortalised (P19) and primary | 5000 | 6 h | Mitochondrial respiration, glutathione metabolism, enzyme activity, protein expression | Reactive Oxygen Species (ROS): Increased in undifferentiated but not differentiated P19 cells  Superoxide Dismutase (SOD): Decreased activity with VPA exposure; Protein expression remains unchanged  SOD2 acetylation: VPA acetylates SOD2 at K68 in undifferentiated cells  Effects in gestational day 8 mice embryos: Decreased SOD and SOD2 activity; Increased AcK68 SOD2  VPA mechanism: Likely promotes changes to oxidant milieu indirectly, not acting as a direct oxidant |
| **Rout** | **2009** | Rat | Immortalised (PC12) | 25,000 or 50,000 | 4 hr | Gene and protein expression | GATA binding: Increased with 25,000 μM or 50,000 μM VPAGATA3 role: Involved in brain development, cell differentiation, and neurotransmitter synthesis Expressed exclusively in neurons in postnatal mice, mainly in serotonergic and GABAergic neurons Disturbances associated with Autism Spectrum Disorder (ASD)Inflammation and GATA-3: Increased pro-inflammatory cytokines and GATA-3 activity co-detected in infection Potentially increases risk of abnormal brain developmentPotential link to ASD symptoms: Enhanced GATA-3 activity may cause excessive differentiation in neurons involved in sound perception, which possibly explains noise sensitivity in individuals with ASD Excessive differentiation of serotonergic and dopaminergic neurons mediated by GATA-3 may cause ASD |
| **Bollino** | **2015** | Rat | Immortalised (PC12) | 50, 200, 500, 1000, 5000 | 3 or 5 days | Cell viability, survival pathway expression | Cell death threshold: 35% cell death (65% cell viability) above 1000μM VPA  Calpain involvement: VPA induces cell death via calpain activation; PD150696 (calpain inhibitor) significantly prevented cell death in VPA-treated primary neurons  Cell death pathway: Atypical calpain-dependent necroptosis; Involves downstream activation of JNK1; Increased expression of RIP-1; Accompanied by AIF cleavage, mitochondrial release, and nuclear translocation |
| **Dedoni** | **2020** | Human and mouse | Immortalised (SH-SY5Y and LAN-1) and primary | 1000 | 24 hr | Gene & protein expression, cell viability | Upregulation of p75NTR and sortilin: Observed in SH-SY5Y and LAN-1 human neuroblastoma cells; Increased cell surface expression of both proteins  Effects on proNGF-induced signaling: Enhances formation of p75NTR/sortilin complex; Activates JNK signaling  Apoptotic effects: Induces neuroblastoma cell apoptosis; Apoptosis is potentiated by proNGF  Effects on mouse cerebellar granule cells: Enhances p75NTR and sortilin expression; Promotes proNGF-induced apoptosis |
| **Kakumoto** | **2021** | Human | Immortalised (SH-SY5Y) | 100, 300, 500, 1000 | 72 hr | Antiproliferation, cell morphology, gene expression | Gene expression changes   SCN1A (sodium channel gene):  Increased at 24, 48, 72 hr with 300-1000 μM VPA; Fetal overexpression could affect neurodevelopment  SLC7A5 (folate transporter):  Increased at 72 hr with 1000 μM VPA; Short culture time likely prevented folate transport effects |
| **Hinojosa** | **2023** | Human | Immortalised (SH-SY5Y) | 0.0001 to 10,000 | 6 days | Gene expression, cell viability, neurite outgrowth | Neurite Outgrowth and Cell Viability: Attenuated neurite outgrowth (dose-dependent), significant at 254 μM; No significant effect on cell viability until 660 μM+  Gene Dysregulation: 16 genes affected - 4 upregulated, 12 downregulated  Axonal Outgrowth Genes Affected: NTNG2, NRXN1, SEMA5A |
| **Chaudhary** | **2021** | Human and Rat | Immortalised (SH-SY5Y) and primary | 1000, 5000, 10000 | 24 hr | Cell morphology, cell viability, cytotoxicity, oxidant production, protein expression | SH-SY5Y cell viability: Decreased by 45% at 10,000 μM VPA VPA-induced cytotoxicity mechanisms: Mediated by oxidative stress; Associated with induction of apoptosis Cellular apoptosis: Increased in both brain regions (TUNEL assay); Melatonin decreased apoptosis Inflammatory markers: VPA increased TNF-α and IL-6 expression in both brain regions; Melatonin decreased these inflammatory markers |
| **Schilter** | **1995** | Rat | Primary | 139, 277, 555 | 8 or 9 days | Enzyme activity | Valproate increased ChAT, GAD and CNP activity at 277 or 555 uM in mature rat brain cell aggregate cultures, possibly indicating differentiation enhancing effects |
| **Fennrich** | **1998** | Rat (Wistar) | Primary | 50, 500, 1000, 2500, 5000 | 14 days | Cell morphology, gene and protein expression, cell viability | Astrocytic changes: Reduced process length; Misrouting of processes; No morphological changes in hilus region stellate astrocytes  At 0.5 mM for 2 weeks: Distorted and randomized spatial orientation of glial cell processes; Less frequent perpendicular orientation to stratum pyramidale in CA1; Hypertrophic somata with irregular morphology  Astrocyte morphology significantly changes at 2500 or 5000 uM VPA and cell integrity reduces. This can prevent proper networking of neurons. |
| **Hogberg** | **2010** | Rat | Primary | 130, 200, 300 | 1, 4, 8, 12 days | Cell viability, gene and protein expresison | Cell viability: Decreased at 130, 200, and 300 μM by day 8 and day 12Cell population changes: Significant astrocyte proliferation at all VPA concentrations; Significant reduction in neuron numbersNeurotransmitter receptors: GABA_A: Downregulated by VPA (130 μM) earlier than NMDA; Possible main mechanism of VPA toxicity due to GABA release modification |
| **Wang** | **2011** | Human | Primary | 0, 250, 500, 1000 | 7 days | Protein expression, apoptosis | VPA selectively induced apoptosis in neurons, not astrocytes  Astrocyte morphology changes: Longer processes; Cytoplasm shrinkage; Stronger GFAP immunoreactivity  TNF-α involvement: Significantly increased in mixed culture supernatants after 5-7 days; Correlated with VPA concentration  Astrocytes activated by VPA to release TNF-α  TNF-α caused dose-dependent neuronal apoptosis  Blocking TNF-α with antibodies reduced neuronal apoptosis |
| **Chaudhary** | **2012** | Rat (Wistar) | Primary | 0.69 to 714 | 3 h | Lipid peroxidation, oxidative damage, enzyme activity, glutathione metabolism | Lipid peroxidation (LPO):Induced at ≥0.5 mg VPA Oxidative damage: Increased protein carbonyl (PC) content  Non-enzymatic antioxidants: NP-SH (non-protein sulfhydryl) diminished at ≥0.5 mg VPA; GSH (glutathione) diminished at ≥0.1 mg VPA Enzymatic antioxidant activity: SOD (superoxide dismutase) decreased; XO (xanthine oxidase) increased |
| **Morte** | **2013** | Rat (Wistar) | Primary | 10, 30, 100, 300, 500, 1000, 3000 | 24 hr | Cell viability, protein staining | Apoptotic markers: Increased expression of cleaved caspase-3 and cleaved PARP at 3000 μM VPA  Cell death pathway activation was confirmed despite lack of observable cell death. Possible explanations: a. Caspase-3 cleavage occurs early in cell death cascade; b. Caspase-3 cleavage necessary but not sufficient for cell death; c. Potential activation of cell survival pathways  Neuroprotective effects: Activation of Erk, Akt, and JNK signaling pathways consistent with known neuroprotective effects of VPA in mature neurons |
| **Zieminska** | **2016** | Rat (Wistar) | Primary | 10 to 5000 | 24 hr | Gene & protein expression | SNAP-25 protein expression: 27% reduction observed with 5000 μM of VPA SNAP-25 function: Involved in presynaptic intracellular vesicular trafficking and exocytosis; Reduction in protein associated with hyperactivity and cognitive abilities in neurodevelopmental disorders |
| **Al-Rubai** | **2017** | Human | Primary | 250, 500, 750, 1000, 1500, 2000, and 2500 | 6 or 10 days | Cell viability, cell migration, neuronal process length, protein expression | Protein expression:  GFAP (astrocyte marker): Decreased at highest doses  Significant effect on astrocytes even at therapeutic doses |
| **Kim** | **2020** | Rat (Sprague-Dawley) | Primary | 600, 1200, 2500, 5000 | 24 hr | Gene and protein expression, cell viability | T-type calcium channels: Increased in embryonic cortices and NPCs after prenatal VPA exposure; CaV3.1 upregulated via epigenetic expression  Epigenetic mechanism: VPA acetylates histone H3 bound to Cacna1g promoter in cortices and NPCs; CaV3.1 identified as an epigenetic target of VPA during embryonic cortical development  Calcium influx: Increased activity-dependent calcium influx in differentiated NPCs exposed to VPA  NPC proliferation: Increased by T-type calcium channels; VPA increased the number of viable NPCs; T-type calcium channel blockers prevented VPA-induced NPC proliferation  Neurodevelopmental implications: T-type calcium channels implicated in autism spectrum disorders and absence seizures; Gain-of-function mutations associated with severe motor and cognitive impairments and epilepsy |
| **Fujiki** | **2013** | Mouse | Stem (E14TG2a) | 100, 500, 3000, 20000 | 3, 6, 9, 12, 15, or 24 h | Protein expression, cell viability | Apoptosis in NPCs: Induced by VPA from 100-20000 μM; This range includes therapeutic concentrations  Effect on mature neurons: No apoptosis observed in mature glutamatergic neurons  Histone acetylation: Enhances histone H3 acetylation in NPCs; Also enhances H3 acetylation in neurons, but less efficiently  Implications: Dysfunction of histone deacetylases (HDACs) during pregnancy may increase risk of congenital malformations |
| **Miranda** | **2018** | Human | Stem (F002.1 A.13) | 0, 10, 100, 300, 500, 1000 | 9 days | Gene and protein expression, cell morphology | Gene expression: Normal neural differentiation path up to 0.3 mM VPA; Altered gene expression at 0.5 mM VPA; e.g. High Sox1 expression from day 9 to 19 at 0.5 mM VPA |
| **Chanda** | **2019** | Human and Mouse | Stem (H1 and H9) | 1000 | 72-96 hr | Gene and protein expression | VPA affects early development but not mature neurons. VPA inhibits HDAC and GSK-3-mediated maturation. VPA suppressed MARCKSL1, knocking down MARCKSL1 induces defects, overexpressing MARCKSL1 rescues VPA-induced impairments. |
| **Schulpen (a)** | **2015** | Human and mouse | Stem (H9 and ES-D3) | mESC = 15, 60, 250, 1000. hESC = 100, 300, 1000 | 24 h (for both) | Gene expression | Potential biomarkers for species-independent compound-mediated DNT:   143 genes regulated by both time and VPA exposure in both assays  Enriched GO terms: regulation of cell proliferation and regeneration  Important genes involved in neural and general development processes  25 candidate marker genes for DNT:   Neural-associated: TUBB3, HOXA1, ADM, LHX1, EPHA2, GAP43, STMN2, MAP2, NEFL, CHRNA3, METRN, DNMT3B, T, NODAL, INA, ZIC3, CTGF  General development: FGF8, MYC, TXNIP, ANXA1, CTGF, COL1A2, MLLT3, RHOU  GAP43, INA, and LHX previously published as candidate markers for DNT in vitro  Species differences: Gene expression patterns and profiles differ between hESCs and mESCs; Suggests mouse models may be less translatable to human clinical outcomes |
| **Shinde** | **2016** | Human | Stem (H9 and foreskin and IMR90) | 1000 | 14 days | Gene expression | Developmental impact: VPA disturbs expression of genes involved in late, specific differentiation processes of somatic cells  Specific gene effects: a. Downregulated by VPA: DOK6: Knockdown causes decreased neurite outgrowth; BCL2: Knockout in mice leads to progressive degeneration of facial neurons  b. Upregulated by VPA: CLDN10; PRKCB |
| **Colleoni** | **2014** | Human | Stem (H9 and HUES1) | 3, 30, 300, 1050 or 1250 | 9 days | Gene expression and GO term analysis | 36 Upregulated genes Notable genes: EPHA7: Expressed on edges of neural tube; TEX15: Expressed on midline of neural plate; Both associated with neural tube defects  55 Downregulated genes Affected biological processes: Nervous system development, Cellular adhesion, Cell migration, Regulation of transcription, Regulation of apoptosis |
| **West** | **2010** | Human | Stem (H9) | 1154 | 4 days | Cell viability, enzyme activity, metabolism | Cell viability: Increased by VPA  Asymmetric dimethylarginine (ADMA): Significantly decreased with VPA exposure; ADMA is an inhibitor of nitric oxide synthase (NOS)  Arginine/ADMA ratio: Fold change of 2.11 with VPA exposure; Predicted to be teratogenic |
| **Krug** | **2013** | Human | Stem (H9) | 500, 600, 1050, 1210 or 2000 | 6, 9 or 14 days | Gene expression, cytotoxicity, pharmacokinetics | Gene regulation patterns:   Down-regulated genes: RNA processing, chromatin modification/histone acetylation  Consistent with VPA's known histone deacetylase inhibitor (HDACi) activity  Enriched GO terms: neural tube formation, neuron development, embryonic morphogenesis |
| **Palmer** | **2013** | Human | Stem (H9) | 4 to 30000 | 72 hr | Metabolism, cell viability | Ornithine levels: Decreased with VPA exposure; Ornithine/citrulline (o/c) ratio decreased Timing of effects: Ornithine decrease observed before cytotoxicity Teratogenic concentrations: O/c ratio becomes teratogenic at 90.8 μM VPA; Cell viability becomes teratogenic at 1113.7 μM VPA Maximum concentration (Cmax): 1000 μM for VPA |
| **Waldmann** | **2014** | Human | Stem (H9) | 25 to 1000 | 6 days | Gene expression | Cytotoxicity: Non-cytotoxic up to 550 μM; 20-30% decrease in viability at 800 and 1000 μM  Gene regulation patterns: Gradual increase in up/down-regulated probe sets (PS) from 350 to 1000 μM  Two subgroups: genes regulated only at higher concentrations and genes regulated concentration-dependently  Biological processes affected:  Key processes altered at low and medium concentrations, and a few additional processes changed at high (cytotoxic) concentrations  Developmental disturbances, cell migration, and down-regulation of neuronal pathways observed between 150 and 550 μM  Concentration-dependent effects: No fold change at 25 μM, suggesting a threshold mechanism |
| **Schulpen (b)** | **2015** | Human | Stem (H9) | 100, 330, 1000 | 1 or 7 days | Gene expression | Gene expression changes: 3696 and 5233 genes differentially expressed after 1 or 7 days of VPA exposure, respectivelyEnriched GO terms after VPA exposure: 'Ion transport'; 'Synapse'; 'Axon'; 'Calcium ion binding'; 'Chromatin modification'Pathways affected after 1 day of VPA exposure: 61 significantly regulated pathways; 8 involved in mechanism of action, neurodevelopment, and toxicityDevelopmental GO terms: 'Embryonic and Tissue Morphogenesis' (overlapping between VPA and CBZ after 7 days); 'Axonogenesis' and 'Neuron Projection Morphogenesis' (overlapping between VPA days 1 and 7)Signaling pathways affected: Wnt signaling: Downregulation of Tcf/Lef genes (impacts stem cell self-renewal, mesoderm, neuroectoderm, and body axis formation) TGF-β signaling: Affects cell proliferation, differentiation, and migration ERK pathway: Activated by VPA (impacts cell growth, proliferation, survival, neuronal signal transduction, and neuron maturation) |
| **Schulpen (c)** | **2015** | Human | Stem (H9) | 3.3, 100, 330, 1000, 2000 | 5 or 7 days | Gene and protein expression, cell viability | Gene expression: Significant concentration-dependent regulation Stem cell-related genes: Decreased expression of Pou5F1 and Nanog Neural development-related genes: Down-regulated: βIII-tubulin, Neurog1, Reelin; Up-regulated: Map2, Mapt  Concentration-dependent effects:   Lower concentrations: Neuroprotective, induces differentiation and neural outgrowth  Higher concentrations: Inhibits βIII-tubulin and Neurog1 expression, potentially neurotoxic  Reelin expression inhibited across all concentrations |
| **Waldmann** | **2017** | Human | Stem (H9) | 600, 1000, 3000, 4000, 5000 | 11 days or 15 days | Gene expression, cell viability, enzyme activity | Gene expression changes: 396 upregulated and 405 downregulated cytotoxicity genes within 48 hours  Proposed mechanisms of action: Direct alteration of epigenetic state, leading to large transcriptome alterations at non-cytotoxic conditions  Implications:   VPA's effects on gene expression consistent with its role as an HDAC inhibitor  Transcriptome alterations may precede and contribute to cytotoxicity  Difficulty in interpreting down-regulated genes as cytotoxicity markers |
| **Zang** | **2022** | Human | Stem (H9) | 100, 250, 500 | 12 days | Cell viability, proliferation & expansion | VPA effects on organoid development: Inhibited proliferation and expansion of human dorsal forebrain organoids; Reduced neural progenitor cells by day 56  Cortical lamination: VPA exposure disorganized cortical lamination; Caused loss of upper layer neurons  Outer radial glia (oRG)-like cells: VPA exposure reduced oRG-like cell production |
| **de Leeuw** | **2022** | Human | Stem (H9) | 6 or 250 | 5 or 10 days | Cell viability, cell morphology, gene and protein expression | Increased protein expression:   NPCs: TUBB3 and MAP2  Synaptic markers: SYNPR and PSD95 (encoded by DLG4)  Vesicle marker: VGLUT2 (encoded by SLC17A6) |
| **Pistollato** | **2020** | Human | Stem (IMR-90) | 10, 39, 156, 625, 2500, 10000 | 3 days or 14 days | Cell viability, protein expression | Neurite morphology changes after 3-day treatment:   IC5 (0.07 mM) VPA: Increased number of neurites per neuron  IC20 (0.21 mM) VPA:  a. Increased neurite length  b. Increased number of branch points |
| **Adler** | **2008** | Human | Stem (SA002) | 4500 | 10 days | Cell viability | EC50 for ATP assay: Human embryonic stem cells (hES): 3255 ± 1136 μM Human embryonic stem cell-derived mesenchymal progenitors (hES MP): 1111 ± 210 μM Human foreskin fibroblasts (hFF): 2296 ± 564 μMEC50 for RES (resazurin) assay: Human embryonic stem cells (hES): 2455 ± 1052 μM Human embryonic stem cell-derived mesenchymal progenitors (hES MP): 707 ± 213 μM Human foreskin fibroblasts (hFF): 1463 ± 216 μM |
| **Konala** | **2021** | Human | Stem (TC-1133) | 1000 to 80000 | 15 days | Cell viability, apoptosis, gene and protein expression | Effects on stem cell markers: Increased OCT4 and SOX2 expression  Histone deacetylase (HDAC) inhibition: Specific inhibition of class I HDACs by VPA confirmed  Study limitations: Used VPA concentrations far above therapeutic range; Examined effects on all three germ layers, not just ectoderm (neural) |
| **Smirnova** | **2014** | Mouse | Stem (W4) | 300 | 16 days / until sample is taken | Gene and protein expression | Toxicity and general effects: EC10: 314 mM; BIII-tubulin expression reduced up to 65% of control  Specific miRNA changes:   Upregulated: Myogenic miRNAs (mir-206, mir-133a,b, mir-10a, mir-143/145 cluster, mir-214, mir-322, mir-199a)  Downregulated: Neurogenesis-related miRNAs (mir-137, mir-128, mir-124a, mir-326, mir-7)  mir-206: Most strongly induced (up to 100-fold)  mir-137: Most VPA-sensitive (3.8-fold downregulated)  Gene expression changes:   377 genes significantly affected: 267 upregulated (up to 7-fold), 110 downregulated (2- to 5-fold)  Upregulated: Myogenic genes (Actc1, calponin, myosin light chain, asporin, decorin), Hox genes, Twist1  Downregulated: Neural factors (Otx1, Otx2, Zic3, 4, 5)  Signaling pathways affected: Wnt/β-catenin pathway components perturbed;  Changes in VPA-sensitive genes involved in neural tube closure and defects  Implications: VPA activates myogenesis during neural differentiation of mESCs through upregulation of myogenic miRNAs and regulatory factors |

**References**

1. Attoff K, Gliga A, Lundqvist J, Norinder U, Forsby A. Whole genome microarray analysis of neural progenitor C17.2 cells during differentiation and validation of 30 neural mRNA biomarkers for estimation of developmental neurotoxicity. PloS one. 2017;12(12):e0190066.

2. Martin ML, Breen KC, Regan CM. Perturbations of cellular functions integral to neural tube formation by the putative teratogen sodium valproate. Toxicology in Vitro. 1988 Jan;2(1):43–8.

3. Regan CM, Gorman AM, Larsson OM, Maguire C, Martin ML, Schousboe A, et al. In vitro screening for anticonvulsant-induced teratogenesis in neural primary cultures and cell lines. Int J Dev Neurosci. 1990;8(2):143–50.

4. Radio NM, Breier JM, Shafer TJ, Mundy WR. Assessment of chemical effects on neurite outgrowth in PC12 cells using high content screening. Toxicol Sci. 2008;105(1):106–18.

5. Jergil M, Kultima K, Gustafson AL, Dencker L, Stigson M. Valproic Acid–Induced Deregulation In Vitro of Genes Associated In Vivo with Neural Tube Defects. Toxicological Sciences. 2009 Mar;108(1):132–48.

6. Lamparter CL, Philbrook NA, Winn LM. Valproic acid increases NF-κB transcriptional activation despite decreasing DNA binding ability in P19 cells, which may play a role in VPA-initiated teratogenesis. Reproductive Toxicology. 2017 Dec;74:32–9.

7. Hansen JM, Lucas SM, Ramos CD, Green EJ, Nuttall DJ, Clark DS, et al. Valproic acid promotes SOD2 acetylation: a potential mechanism of valproic acid-induced oxidative stress in developing systems. Free Radical Research. 2021 Dec 2;55(11–12):1130–44.

8. Rout UK, Clausen P. Common increase of GATA-3 level in PC-12 cells by three teratogens causing autism spectrum disorders. Neuroscience Research. 2009 Jun;64(2):162–9.

9. Bollino D, Balan I, Aurelian L. Valproic acid induces neuronal cell death through a novel calpain‐dependent necroptosis pathway. Journal of Neurochemistry. 2015 Apr;133(2):174–86.

10. Dedoni S, Marras L, Olianas MC, Ingianni A, Onali P. Valproic acid upregulates the expression of the p75NTR/sortilin receptor complex to induce neuronal apoptosis. Apoptosis. 2020 Oct;25(9–10):697–714.

11. Kakumoto M, Shimokawa K, Ueshima S, Hira D, Okano T. Effects of antiepileptic drugs’ administration during pregnancy on the nerve cell proliferation and axonal outgrowth of human neuroblastoma SH-SY5Y nerve cells. Biochemical and Biophysical Research Communications. 2021 May;554:151–7.

12. Hinojosa MG, Johansson Y, Cediel-Ulloa A, Ivanova E, Gabring N, Gliga A, et al. Evaluation of mRNA markers in differentiating human SH-SY5Y cells for estimation of developmental neurotoxicity. NeuroToxicology. 2023 Jul;97:65–77.

13. Chaudhary S, Sahu U, Parvez S. Melatonin attenuates branch chain fatty acid induced apoptosis mediated neurodegeneration. Environmental Toxicology. 2021 Apr;36(4):491–505.

14. Schilter B, Noldner M, Chatterjee S, Honegger P. Anticonvulsant drug toxicity in rat brain cell aggregate cultures. TOXICOLOGY IN VITRO. 1995;9(4):381–6.

15. Fennrich S, Ray D, Nau H, Schlosshauer B. Radial astrocytes: Toxic effects induced by antiepileptic drug in the developing rat hippocampus in vitro. European Journal of Cell Biology. 1998 Oct;77(2):142–50.

16. Hogberg HT, Kinsner-Ovaskainen A, Coecke S, Hartung T, Bal-Price AK. mRNA Expression is a Relevant Tool to Identify Developmental Neurotoxicants Using an In Vitro Approach. Toxicological Sciences. 2010 Jan;113(1):95–115.

17. Wang C, Luan Z, Yang Y, Wang Z, Cui Y, Gu G. Valproic acid induces apoptosis in differentiating hippocampal neurons by the release of tumor necrosis factor-α from activated astrocytes. Neuroscience Letters. 2011 Jun;497(2):122–7.

18. Chaudhary S, Parvez S. An in vitro approach to assess the neurotoxicity of valproic acid-induced oxidative stress in cerebellum and cerebral cortex of young rats. Neuroscience. 2012;225(nzr, 7605074):258–68.

19. Morte MI, Carreira BP, Falcão MJ, Ambrósio AF, Soares-da-Silva P, Araújo IM, et al. Evaluation of neurotoxic and neuroprotective pathways affected by antiepileptic drugs in cultured hippocampal neurons. Toxicology in Vitro. 2013 Dec;27(8):2193–202.

20. Zieminska E, Lenart J, Lazarewicz JW. Select putative neurodevelopmental toxins modify SNAP-25 expression in primary cultures of rat cerebellar granule cells. Toxicology. 2016 Aug;370:86–93.

21. Al-Rubai A, Wigmore P, Pratten MK. Evaluation of a Human Neural Stem Cell Culture Method for Prediction of the Neurotoxicity of Anti-epileptics. Altern Lab Anim. 2017 May;45(2):67–81.

22. Kim JW, Oh HA, Kim SR, Ko MJ, Seung H, Lee SH, et al. Epigenetically Upregulated T-Type Calcium Channels Contribute to Abnormal Proliferation of Embryonic Neural Progenitor Cells Exposed to Valproic Acid. Biomolecules & Therapeutics. 2020 Sep 1;28(5):389–96.

23. Fujiki R, Sato A, Fujitani M, Yamashita T. A proapoptotic effect of valproic acid on progenitors of embryonic stem cell-derived glutamatergic neurons. Cell Death Dis. 2013 Jun 20;4(6):e677–e677.

24. Miranda CC, Fernandes TG, Pinto SN, Prieto M, Diogo MM, Cabral JMS. A scale out approach towards neural induction of human induced pluripotent stem cells for neurodevelopmental toxicity studies. Toxicology Letters. 2018 Sep;294:51–60.

25. Chanda S, Ang CE, Lee QY, Ghebrial M, Haag D, Shibuya Y, et al. Direct Reprogramming of Human Neurons Identifies MARCKSL1 as a Pathogenic Mediator of Valproic Acid-Induced Teratogenicity. Cell Stem Cell. 2019 Jul;25(1):103-119.e6.

26. Schulpen SHW, Theunissen PT, Pennings JLA, Piersma AH. Comparison of gene expression regulation in mouse- and human embryonic stem cell assays during neural differentiation and in response to valproic acid exposure. Reproductive Toxicology. 2015 Aug;56:77–86.

27. Shinde V, Perumal Srinivasan S, Henry M, Rotshteyn T, Hescheler J, Rahnenführer J, et al. Comparison of a teratogenic transcriptome-based predictive test based on human embryonic versus inducible pluripotent stem cells. Stem Cell Res Ther. 2016 Dec;7(1):190.

28. Colleoni S, Galli C, Gaspar JA, Meganathan K, Jagtap S, Hescheler J, et al. A comparative transcriptomic study on the effects of valproic acid on two different hESCs lines in a neural teratogenicity test system. Toxicology Letters. 2014 Nov;231(1):38–44.

29. West PR, Weir AM, Smith AM, Donley ELR, Cezar GG. Predicting human developmental toxicity of pharmaceuticals using human embryonic stem cells and metabolomics. Toxicology and Applied Pharmacology. 2010 Aug;247(1):18–27.

30. Krug AK, Kolde R, Gaspar JA, Rempel E, Balmer NV, Meganathan K, et al. Human embryonic stem cell-derived test systems for developmental neurotoxicity: a transcriptomics approach. Archives of Toxicology. 2013;87(1):123–43.

31. Palmer JA, Smith AM, Egnash LA, Conard KR, West PR, Burrier RE, et al. Establishment and assessment of a new human embryonic stem cell-based biomarker assay for developmental toxicity screening. Birth defects research Part B, Developmental and reproductive toxicology. 2013;98(4):343–63.

32. Waldmann T, Rempel E, Balmer NV, Konig A, Kolde R, Gaspar JA, et al. Design principles of concentration-dependent transcriptome deviations in drug-exposed differentiating stem cells. Chemical Research in Toxicology. 2014;27(3):408–20.

33. Schulpen SHW, De Jong E, De La Fonteyne LJJ, De Klerk A, Piersma AH. Distinct gene expression responses of two anticonvulsant drugs in a novel human embryonic stem cell based neural differentiation assay protocol. Toxicology in Vitro. 2015 Apr;29(3):449–57.

34. Schulpen SHW, Pennings JLA, Piersma AH. Gene expression regulation and pathway analysis after valproic acid and carbamazepine exposure in a human embryonic stem cell-based neurodevelopmental toxicity assay. Toxicological Sciences. 2015;146(2):311–20.

35. Waldmann T, Grinberg M, Konig A, Rempel E, Schildknecht S, Henry M, et al. Stem Cell Transcriptome Responses and Corresponding Biomarkers That Indicate the Transition from Adaptive Responses to Cytotoxicity. Chemical Research in Toxicology. 2017;30(4):905–22.

36. Zang Z, Yin H, Du Z, Xie R, Yang L, Cai Y, et al. Valproic acid exposure decreases neurogenic potential of outer radial glia in human brain organoids. Front Mol Neurosci. 2022 Nov 29;15:1023765.

37. De Leeuw VC, Van Oostrom CTM, Wackers PFK, Pennings JLA, Hodemaekers HM, Piersma AH, et al. Neuronal differentiation pathways and compound-induced developmental neurotoxicity in the human neural progenitor cell test (hNPT) revealed by RNA-seq. Chemosphere. 2022 Oct;304:135298.

38. Pistollato F, de Gyves EM, Carpi D, Bopp SK, Nunes C, Worth A, et al. Assessment of developmental neurotoxicity induced by chemical mixtures using an adverse outcome pathway concept. Environmental health : a global access science source. 2020;19(1):23.

39. Adler S, Lindqvist J, Uddenberg K, Hyllner J, Strehl R. Testing Potential Developmental Toxicants with a Cytotoxicity Assay Based on Human Embryonic Stem Cells. Altern Lab Anim. 2008 May;36(2):129–40.

40. Konala VBR, Nandakumar S, Surendran H, Datar S, Bhonde R, Pal R. Neuronal and cardiac toxicity of pharmacological compounds identified through transcriptomic analysis of human pluripotent stem cell-derived embryoid bodies. Toxicology and Applied Pharmacology. 2021 Dec;433:115792.

41. Smirnova L, Block K, Sittka A, Oelgeschlager M, Seiler AEM, Luch A. MicroRNA profiling as tool for in vitro developmental neurotoxicity testing: the case of sodium valproate. PloS one. 2014;9(6):e98892.
